# No Impact of Body Mass Index on Outcome in Stroke Patients Treated with IV Thrombolysis BMI and IV Thrombolysis Outcome

**DOI:** 10.1371/journal.pone.0164413

**Published:** 2016-10-11

**Authors:** Meret Branscheidt, Juliane Schneider, Patrik Michel, Elissavet Eskioglou, Georg Kaegi, Robert Stark, Urs Fischer, Simon Jung, Marcel Arnold, Maria Wertli, Ulrike Held, Susanne Wegener, Andreas Luft, Hakan Sarikaya

**Affiliations:** 1 Department of Neurology, University Hospital of Zurich, Switzerland; 2 Department of Physical Medicine and Rehabilitation, Johns Hopkins University, Baltimore, Maryland, United States of America; 3 Department of Neurology, Centre Hospitalier Universitaire Vaudois and University of Lausanne, Switzerland; 4 Department of Neurology, Kantonsspital St. Gallen, Switzerland; 5 Department of Neurology, University Hospital Bern, Switzerland; 6 Horten Centre, University of Zurich, Switzerland; 7 Department of Internal Medicine, University Hospital Bern, Switzerland; Massachusetts General Hospital, UNITED STATES

## Abstract

**Background and Purpose:**

The impact of excess body weight on prognosis after stroke is controversial. Many studies report higher survival rates in obese patients (“obesity paradox”). Recently, obesity has been linked to worse outcomes after intravenous (IV) thrombolysis, but the number and sample size of these studies were small. Here, we aimed to assess the relationship between body weight and stroke outcome after IV thrombolysis in a large cohort study.

**Methods:**

In a prospective observational multicenter study, we analyzed baseline and outcome data of 896 ischemic stroke patients who underwent IV thrombolysis. Patients were categorized according to body mass index (BMI) as underweight (<18.5 kg/m^2^), normal weight (18.5–24.9 kg/m^2^), overweight (25–29.9 kg/m^2^), obese (30–34.9 kg/m^2^) or severely obese (>35 kg/m^2^). Using uni- and multivariate modeling, we assessed the relationship of BMI with favorable outcome (defined as modified Rankin Scale 0 or 1) and mortality 3 months after stroke as well as the occurrence of symptomatic intracerebral hemorrhages (sICH). We also measured the incidence of patients that had an early neurological improvement of >40% on the National Institutes of Health Stroke Scale (NIHSS) after 24 hours.

**Results:**

Among 896 patients, 321 were normal weight (35.8%), 22 underweight (2.5%), 378 overweight (42.2%), 123 obese (13.7%) and 52 severely obese (5.8%). Three-month mortality was comparable in obese vs. non-obese patients (8.1% vs. 8.3%) and did not differ significantly among different BMI groups. This was also true for favorable clinical outcome, risk of sICH and early neurological improvement on NIHSS at 24 hours. These results remained unchanged after adjusting for potential confounding factors in the multivariate analyses.

**Conclusion:**

BMI was not related to clinical outcomes in stroke patients treated with IVT. Our data suggest that the current weight-adapted dosage scheme of IV alteplase is appropriate for different body weight groups, and challenge the existence of the obesity paradox after stroke.

## Introduction

Stroke and obesity are both characterized by increasing incidence worldwide, causing huge socio-economic costs [1]. While obesity is an established risk factor for stroke occurrence, its impact on outcome after stroke is controversial. Many studies suggest survival benefit and better clinical outcome in obese stroke patients as compared to their normal weight counterparts [2–4]. This observation has already been described in many other conditions such as heart failure, and is termed as “obesity paradox” [4]. The relationship between obesity and stroke outcome after intravenous thrombolysis (IVT) is less clear due to scarce number of studies with small sample size and contradictory results. This issue is of clinical relevance, as obesity especially affects younger people with increase of stroke risk and thus higher probability for IVT. Therefore, we aimed to investigate clinical outcomes after IVT in a large cohort of patients with acute ischemic stroke (AIS) according to body mass index (BMI).

## Methods

As a joint initiative of four Swiss stroke centers (Berne, Zurich, Lausanne, St. Gallen), we performed a large prospective multicenter study to determine the impact of body weight on stroke outcome after IVT (data collection from 2003 to 2014). Patients needed to meet the following two criteria for study inclusion: 1. Treatment with IVT (alteplase) for AIS according to the current guidelines of the European Stroke Organization [5], 2. Availability of body mass index (BMI) at baseline and outcome data at 3 months. Data from individual patients were systematically and prospectively collected in each center by using a standardized form with pre-defined variables as applied in previous studies [6]. Compilation of completed forms from all centers and analyses of the pooled data were performed in the coordinating center in Berne, Switzerland. Detailed data on the number of consecutive patients and study period for each center are available as supplemental material. The study was approved by local ethics committees of the individual study centers in Berne (Kantonale Ethikkomission Bern Nr. 231/2014), St. Gallen (Kantonale Ethikkomission St. Gallen Nr. 280/09), Zurich (Kantonale Ethikkomission Zürich Nr. 2013–0105) and Lausanne (Kantonale Ethikkommision Waadt Nr. 40/07). Patients records were recorded and de-identified prior to analysis. According to the approvals, we did not need informed consent from individuals for retrospective data. All clinical investigations were conducted according to the principles expressed in the Declaration of Helsinki.

The following variables were prospectively collected in all participating centers: age, sex, vascular risk factors according to predefined criteria [7], history of coronary artery disease, antithrombotic medication at stroke onset, initial stroke severity as assessed by the National Institutes of Health Stroke Scale (NIHSS) score [8], stroke etiology according to the Trial of ORG 10172 in Acute Stroke Treatment (TOAST) criteria [9], stroke onset-to-treatment time as well as blood pressure and blood glucose level obtained at admission. All patients treated with IVT were admitted to intermediate or intensive care units for at least 24 hours. All patients underwent brain imaging with computed tomography or magnetic resonance imaging 24 to 48 hours after IVT and in any case of clinical deterioration.

### Body Mass Index Assessment

Body mass index (BMI) was calculated as weight in kilograms divided by height squared in meters. Body weight and height were either measured by nurses or—if not applicable—estimated by the attending stroke physician during the hospital stay. For obesity measures, we adopted the following BMI threshold categories from World Health Organization (WHO): <18.5 kg/m^2^ for underweight, 18.5 to 24.9 kg/m^2^ for normal weight, 25.0 to 29.9 kg/m^2^ for overweight, 30.0 to 34.9 kg/m^2^ for obesity and ≥35 kg/m^2^ for severe obesity [10].

### Assessment of Outcomes

Clinical outcomes were assessed during outpatient visits using the modified Rankin Scale (mRS) score at 3 months [11]. Main outcome measures in this study were (i) favorable outcome (defined as mRS 0 or 1), (ii) good outcome (defined as mRS 0 to 2), (iii) death within 3 months and (iv) symptomatic intracerebral hemorrhage (sICH) according to the definition of the Safe Implementation of Thrombolysis in Stroke-Monitoring Study (SITS-MOST) [12]. In addition, we also used the early neurological improvement by >40% on NIHSS at 24 hours after IVT as a marker of early arterial recanalization [13].

### Statistical Analyses

Descriptive statistics are presented as median and interquartile range (IQR) for the continuous parameters, and number and percentages of total for the categorical variables if not stated differently. Outcome measures were compared with BMI as a categorical variable and as a continuous variable. The category with normal body weight (BMI 18.5 to 24.9 kg/m^2^) served as reference group. We compared demographic and baseline characteristics among different BMI categories by using Fisher exact test or the chi-square test for categorical variables and the Mann-Whitney U test for continuous variables. Univariate analysis of the effect of BMI categories on different outcomes was assessed with logistic regression models, results were presented as estimated odds ratios and corresponding 95% confidence intervals (95% CI). Besides the univariate analysis, adjusted effects of BMI categories on different outcomes were of interest. For the adjustment, the following set of potential confounders was identified a-priori according to the current literature [7]: age, gender, baseline NIHSS score, smoking, history of hypertension, systolic blood pressure at presentation, history of cardiovascular disease, history of diabetes, blood glucose at admission, dyslipidemia, antithrombotic treatment at stroke onset, atrial fibrillation and symptom onset-to-treatment time.

In order to find those variables among the set of potential confounders that influenced the individual outcome, pairwise correlations between the potential confounder and the outcome were assessed. In the final multiple models each confounder with a statistically significant relationship with the outcome was included, in addition to the determinant BMI category [14]. Finally, the interaction of the confounders with the BMI subgroups was tested for significance.

Complete data sets were available for BMI. However, there were missing values in the confounders. Multiple imputation using chained equations and five replications (m = 5) were used for each outcome and corresponding regression analysis. Rubin’s formula was used for the combination of effect estimates and their standard errors from the multiply imputed datasets. We additionally evaluated each confounder for the presence of an interaction term with BMI. The interaction was deleted from the model, if it was not significantly different from zero. Statistical analyses were conducted by using the statistical software R (R Core Team (2015)). R: A language and environment for statistical computing. R Foundation for Statistical Computing, Vienna, Austria. URL https://www.R-project.org) and the R-package “mice” [15]. The level of statistical significance was set to 0.05.

## Results

A total of 896 patients were eligible for this study. Of these, 321 were of normal weight (35.8%, median BMI 23 kg/m^2^), 22 were underweight (2.5%, median BMI 17.7 kg/m^2^), 378 overweight (42.2%, median BMI 27 kg/m^2^), 123 obese (13.7%, median BMI 31 kg/m^2^) and 52 severely obese (5.8%, median BMI 37 kg/m^2^). The main baseline characteristics of the study population are detailed in S1 Table.

Gender was differently distributed among BMI categories with a higher percentage of females in the underweight group (72.7%) as compared to patients with normal weight (46.4%), overweight (33.3%) or obesity (40.0%). In obese and severely obese patients, the prevalence of arterial hypertension, diabetes mellitus, blood glucose at admission and use of antithrombotics at stroke onset were significantly higher as compared to the normal weight group, whereas the baseline NIHSS score tended to be slightly lower. Age and other baseline characteristics were similar in all BMI groups S1 Table.

At 3 months, patients with a BMI <30 kg/m^2^ (non-obese) showed no significant difference for any of the outcome measures in comparison to those with a BMI ≥30 kg/m^2^ (obese), even after adjusting for confounding variables (Table 1). The adjusted analysis for sICH was identical to the univariate analysis because none of the potential confounders was significantly associated with sICH.

**Table 1:** Comparison of Outcomes in patients with BMI ≥30 kg/m^2^ vs. <30 kg/m^2^.

Outcome measures	Unadjusted analysis				Adjusted analysis^†^	
	BMI ≥30 kg/m^2^	BMI <30 kg/m^2^	OR [95% CI]	p value	OR [95% CI]	p value
Favorable outcome (mRS 0–1)	76/161 (47.2%)	278/660 (42.1%)	1.229 [0.869–1.736]	0.243	1.392 [0.926–2.093]	0.105
Good outcome (mRS 0–2)	101/161 (62.7%)	435/660 (65.9%)	0.871 [0.609–1.245]	0.448	0.845 [0.556–1.286]	0.423
Mortality	13/161 (8.1%)	55/660 (8.3%)	0.966 [0.514–1.815]	0.915	1.065 [0.503–2.255]	0.866
sICH	6/170 (3.5%)	18/698 (2.6%)	1.382 [0.540–3.537]	0.500	1.382 [0.540–3.537]	0.500
Early NIHSS improvement	84/156 (53.8%)	324/648 (50.0%)	1.167 [0.822–1.656]	0.389	1.270 [0.876–1.843]	0.199

Compared to the patients with BMI >30 kg/m^2^, patients with BMI <30 kg/m^2^ did not differ in mortality, early NIHSS improvement, favorable or good functional outcome, or risk for sICH. These results did not change after adjusting for known confounders (†): age, gender, baseline NIHSS score, blood glucose at admission, systolic blood pressure at admission, smoking, presence of arterial hypertension, presence of diabetes, dyslipidemia, history of cardiovascular disease, antithrombotic treatment at stroke onset, atrial fibrillation and symptom onset-to-treatment time. Results are presented as odds ratio with 95% confidence intervals. Abbreviations: BMI (body mass index), OR (odds ratio), CI (confidence interval), IQR (inter quartile range), mRS (modified Rankin Scale), sICH (symptomatic intracerebral hemorrhage), NIHSS (National Institute of Health Stroke Scale).

As compared to the normal weight reference group, none of the BMI categories significantly differed with respect to the outcomes clinical recovery, mortality, occurrence of sICH, or early NIHSS improvement (Table 2). In univariate analyses, the following variables were associated with clinical endpoints (favorable outcome, good outcome, mortality): age, stroke severity measured by baseline NIHSS score, diabetes mellitus and baseline blood glucose. In addition, mortality was associated with the presence of atrial fibrillation. Predictors of early NIHSS improvement in univariate analysis were age, diabetes mellitus, baseline blood glucose and systolic blood pressure at admission. For sICH no model could be fitted due to singularity because no sICH was observed in the group of underweight and severely obese patients.

**Table 2:** Univariate outcome analyses according to BMI categories.

Outcome measures	BMI categories n/N (%) OR [95% CI]				
	Normal weight* (n = 321)	Underweight (n = 22)	Overweight (n = 378)	Obesity (n = 123)	Severe Obesity (n = 52)
Favourable outcome (mRS 0–1) ^a^	114/291 (39.2%)	12/21 (57.1%) 2.070 [0.845–5.070]	152/348 (43.7%) 1.204 [0.877–1.653]	56/114 (49.1%) 1.499 [0.969–2.319]	20/47 (42.6%) 1.150 [0.616–2.147]
Good outcome (mRS 0–2) ^b^	194/291 (66.7%)	14/21 (66.7%) 1.000 [0.391–2.559]	227/348 (65.2%)0.938 [0.675–1.303]	75/114 (65.8%) 0.962 [0.609–1.519]	26/47 (55.3%) 0.619 [0.331–1.156]
Mortality ^c^	23/291 (7.9%)	1/21 (4.8%) 0.583 [0.075–4.539]	31/348 (8.9%) 1.139 [0.649–2.002]	8/114 (7.0%) 0.879 [0.381–2.028]	5/47 (10.6%) 1.387 [0.500–3.848]
sICH	11/309 (3.6%)	0/22 (0%) n.a.	7/367 (1.9%) 0.527 [0.202–1.376]	6/120 (5.0%) 1.426 [0.515–3.946]	0/50 (0%) n.a.
Early NIHSS improvement ^d^	135/283 (47.7%)	11/21 (52.4%) 1.206 [0.496–2.930]	178/344 (51.7%) 1.176 [0.858–1.611]	59/106 (55.7%) 1.376 [0.879–2.155]	25/50 (50.0%) 1.096 [0.601–2.000]

^a^ age, baseline NIHSS, diabetes mellitus, gender, blood glucose, atrial fibrillation;

^b^ age, baseline NIHSS, diabetes mellitus, gender, blood glucose;

^c^ age, baseline NIHSS, diabetes mellitus, blood glucose, atrial fibrillation, coronary heart disease, antithrombotic use at baseline;

^d^ age, diabetes mellitus, systolic blood pressure, blood glucose.

Univariate analyses showed no association between different BMI categories and functional outcome, mortality or the occurrence of sICH. BMI categories set in reference to normal weight patients and were defined as follows: <18.5 kg/m^2^ for underweight, 18.5 to 24.9 kg/m^2^ for normal weight, 25.0 to 29.9 kg/m^2^ for overweight, 30.0 to 34.9 kg/m^2^ for obesity and ≥35 kg/m^2^ for severe obesity. Results are presented as odds ratio with 95% confidence intervals. (*) Asterix labels the normal weight reference group. Letters ^a–d^ denote significant associations in univariate analysis (see footnotes). Abbreviations: BMI (body mass index), OR (odds ratio), CI (confidence interval), mRS (modified Rankin Scale), sICH (symptomatic intracerebral hemorrhage), NIHSS (National Institute of Health Stroke Scale), n.a. (not applicable).

After multivariate analyses, the outcomes still did not differ among BMI categories as compared to the normal weight group (Table 3). Although underweight patients showed a trend towards a favorable outcome (mRS 0–1), one has to consider the broad confidence intervals. Age, stroke severity and baseline blood glucose were independent predictors of clinical recovery and mortality, whereas systolic blood pressure and blood glucose at admission were significantly associated with early NIHSS improvement.

**Table 3:** Multivariate outcome analyses according to BMI categories.

Outcome measures	BMI categories OR [95% CI]			
	Underweight vs. normal weight	Overweight vs. normal weight	Obesity vs. normal weight	Severe Obesity vs. normal weight
Favorable outcome (mRS 0–1) ^a^	2.847 [1.000–8.112]	1.094 [0.756–1.583]	1.569 [0.942–2.614]	1.375 [0.660–2.867]
Good outcome (mRS 0–2) ^b^	1.084 [0.361–3.256]	0.791 [0.537–1.166]	0.856 [0.503–1.459]	0.549 [0.263–1.144]
Mortality ^c^	0.441 [0.046–4.270]	1.341 [0.684–2.629]	1.081 [0.400–2.919]	1.605 [0.469–5.486]
Early NIHSS improvement ^d^	1.120 [0.445–2.820]	1.160 [0.835–1.611]	1.419 [0.886–2.273]	1.301 [0.685–2.473]

^a^ baseline NIHSS (p<0.001), age (p = 0.008), blood glucose (p = 0.008);

^b^ age (p<0.001), baseline NIHSS (p<0.001);

^c^ age (p<0.001), baseline NIHSS (p<0.001), blood glucose (p = 0.003);

^d^ blood glucose (p = 0.024), systolic blood pressure (p = 0.040).

The multivariate analyses showed no differences between overweight, obese and severely obese patients in reference to normal weight patients regarding functional outcome, mortality or sICH. Results are presented as odds ratio with 95% confidence intervals. Letters ^a–d^ denote significant associations in multivariate analysis. Abbreviations: BMI (body mass index), OR (odds ratio), CI (confidence interval), mRS (modified Rankin Scale), sICH (symptomatic intracerebral hemorrhage), NIHSS (National Institute of Health Stroke Scale).

## Discussion

This multi-center cohort study indicates no significant differences regarding functional outcome after IVT in normal weight patients compared to overweight, obese and underweight patients. Likewise, we found a similar risk of mortality and sICH for each BMI group.

Obesity has become a medical and socio-economic burden with epidemic proportions and is an established risk factor for the incidence of stroke especially in younger patients. Recent studies surprisingly indicate better functional outcome and less mortality rates in stroke patients with excess of body weight as compared to their normal weight counterparts (“obesity paradox”) [4]. Data regarding impact of body weight on outcome after IVT are scarce and showed contradictory results hitherto. Sarikaya et al. reported significantly higher rates of mortality and unfavorable outcome in 53 obese patients as compared to 251 non-obese patients (BMI <30 kg/m^2^), but stated the small size of study cohort study hindering a generalization of the findings and called for larger studies [16]. Seet et al. observed in their study on 169 patients similar rates of functional recovery among obese (n = 54), overweight, and normal weight patients after IVT [17]. In the study by Seo et al. assessing 321 Korean patients treated with IVT, being underweight was independently associated with poorer long-term survival as compared to patients with normal body weight [18]. The present IVT study included more patients than all 3 abovementioned studies together [17,19] and found no evidence for different clinical outcomes among normal weight, underweight, overweight or obese stroke patients. Moreover, we did not observe a difference in outcomes by comparing obese vs. non-obese patients, neither for clinical recovery nor for mortality. In addition, we also assessed clinical outcomes in severely obese patients (BMI > 35 kg/m^2^) separately, but the outcomes were still comparable to normal weight patients. Subsequently, we performed multivariate analyses correcting for potential outcome confounders, but failed to show an association between clinical endpoints and BMI. The same was also true for the risk of sICH, which was not associated with overweight or obesity. Furthermore, we assessed early neurological improvement at 24 hours post-treatment (defined as NIHSS score improvement by ≥ 40% from baseline) as a predictor of recanalization after thrombolysis and again did not find significant differences among the 5 body weight groups. In line with this, the rates of both good clinical outcome (defined as mRS 0 and 1) and favorable clinical outcome (defined as mRS 0 to 2) at 3 months were similarly distributed among the cohorts. Unfortunately, data about radiological recanalization were too sparsely available for a firm analysis.

The number of included patients were appropriate in normal weight, overweight and obese groups (n = 321, 378 and 175, respectively), but probably too low in underweight group (n = 22). Thus, results for the latter body weight group need a cautious interpretation with respect to broad confidence intervals in outcome analyses for this group. Further studies are needed for outcome analyses in these patients, because severe underweight has been shown to be an independent risk factor for intracranial hemorrhages, poor clinical outcome and mortality in acute stroke [20–22]. Underweight is also a main indicator of malnutrition, which has a high prevalence among older patients and significant association with medical complications such as renal insufficiency. Lower glomerular filtration has been suggested to be a better risk indicator for sICH than age in stroke patients treated with IVT [23].

Overall, our data consistently indicate that BMI has no influence on clinical recovery and mortality in patients undergoing IVT for acute ischemic stroke. This finding is of clinical relevance as it indicates that the current weight-adapted dose regimen for IV alteplase as used in daily clinical routine is also appropriate for obese and severely obese patients. Furthermore, our data contradict the popular thesis of “obesity paradox”, which may be an obstacle against weight reduction in obese patients due to false assumptions (such as beneficial effect of obesity for stroke prevention). Several reasons may contribute to these discrepant observations. First, we strictly assessed acute stroke patients treated with IVT with pre-defined outcome analyses at 3 months. In other studies, the frequency of patients treated with thrombolysis was very low or not reported, whereas the follow-up duration ranged from 1 week to 10 years [2,24]. Data in our study were collected prospectively and in a structured way by trained stroke physicians allowing good data quality, whereas data collection was poorly described in other studies or data were derived from national healthcare databases [3,25] As consequence, many studies were not able to correct for relevant outcome predictors such as stroke severity due to data lack of data [2,3,25,26]. Of note, obese stroke patients tend to be younger with lower stroke severity due to higher proportion of lacunar infarcts, which may explain better clinical outcomes in aforementioned studies (selection bias). Thus, the inverse association between BMI and mortality (obesity paradox) disappeared after correcting for stroke severity in some studies. [27,28]. Treatment bias may be another cause of obesity paradox as it has been shown that physicians treat obese patients more aggressively than lean patients due to assumed increase of vascular risk [29]. Publication bias may be a further reason for obesity paradox.

Strengths of our study include (i) the relatively large cohort size of stroke patients addressing the influence of body weight on outcome after IVT, (ii) good quality of clinical data due to prospective and standardized assessments, (iii) separate analysis of all BMI groups from underweight to severely obese patients, and (iv) the use of comprehensive outcome parameters including early neurological improvement at 24 hours as a predictor for recanalization.

Nevertheless, several limitations of our study have to be considered: (i) BMI has been criticized for its low accuracy in measuring obesity especially in older people, while waist-to-hip ratio or waist circumference may be more precise for this purpose [30]. (ii) This is an observational study, thus cautious interpretation is needed especially with respect to causality. To date, randomized controlled trials are lacking in this field. (iii) Body weight or body height were estimated by relatives or nurses in a subgroup of patients, although recent studies indicate that estimated weights to be similar to real values [31]. (iv) Because of the limited numbers of underweight and severely obese patients in our study, results concerning these groups should be interpreted with caution.

## Conclusion

In this study, BMI was not associated with clinical outcomes, risk of sICH or with early neurological improvement after IVT. Our data suggest that the current weight-adapted dosage scheme of alteplase is appropriate for different body weight groups and challenge the existence of obesity paradox after stroke.

## Supporting Information

S1 Table Baseline characteristics according to BMI categories. BMI categories were adopted according to World Health Organization (WHO) guidelines as follows: <18.5 kg/m^2^ for underweight, 18.5 to 24.9 kg/m^2^ for normal weight, 25.0 to 29.9 kg/m^2^ for overweight, 30.0 to 34.9 kg/m^2^ for obesity and ≥35 kg/m^2^ for severe obesity. Body weight groups showed unequal distributions for gender (higher percentage of females in the underweight group), as well as for hypertension and diabetes (higher prevalence in the excess body weight groups). Obese and severely obese patients had increased levels of blood glucose and were more often prescribed antithrombotic drugs. The initial impairment on admission was slightly lower in the high weight groups. Abbreviations: BMI (body mass index), IQR (inter quartile range), NIHSS (National Institute of Health Stroke Scale), CHD (coronary heart disease).
(DOCX)

S2 Table Patient numbers and study periods for each individual center. (DOCX)
