# Supplementary material for: No Impact of Body Mass Index on Outcome in Stroke Patients Treated with IV Thrombolysis BMI and IV Thrombolysis Outcome
Source: PLoS One. 2016 Oct 11;11(10):e0164413. doi: 10.1371/journal.pone.0164413 (PMC5058473; doi:10.1371/journal.pone.0164413)
Supplement: S1 Table — BMI categories were adopted according to World Health Organization (WHO) guidelines as follows: <18.5 kg/m2 for underweight, 18.5 to 24.9 kg/m2 for normal weight, 25.0 to 29.9 kg/m2 for overweight, 30.0 to 34.9 kg/m2 for obesity and ≥35 kg/m2 for severe obesity. Body weight groups showed unequal distributions for gender (higher percentage of females in the underweight group), as well as for hypertension and diabetes (higher prevalence in the excess body weight groups). Obese and severely obese patients had increased levels of blood glucose and were more often prescribed antithrombotic drugs. The initial impairment on admission was slightly lower in the high weight groups. Abbreviations: BMI (body mass index), IQR (inter quartile range), NIHSS (National Institute of Health Stroke Scale), CHD (coronary heart disease). (DOCX) [file pone.0164413.s001.docx]

**Table S1.** Baseline characteristics according to BMI categories

|  | **All** | **BMI (kg/m^2^)** | | | | | **p value** |
| --- | --- | --- | --- | --- | --- | --- | --- |
|  |  |  |  |  |  |  |  |
|  |  | **< 18.5** | **18.5-<25** | **25-<30** | **30-<35** | **≥35** |  |
| **N** | **896** | **22** | **321** | **378** | **123** | **52** |  |
| female *N* (%) | 361 (40.3) | 16 (72.7) | 149 (46.4) | 126 (33.3) | 49 (39.8) | 21 (40.4) | <0.001 |
| Age in years | 72 | 68 | 74 | 72 | 73 | 66 | 0.216 |
| (median [IQR]) | [61.00, 80.00] | [56.75, 80.75] | [61.00, 82.00] | [59.25, 79.00] | [62.00, 79.00] | [61.75, 76.25] |  |
| BMI kg/m^2^ | 26 | 17.65 | 23 | 27 | 31 | 37 | <0.001 |
| (median [IQR]) | [23.00, 29.00] | [15.25, 18.00] | [22.00, 24.00] | [26.00, 28.00] | [30.65, 32.25] | [35.83, 38.00] |  |
| Current smoker (%) | 169 (23.8) | 5 (31.2) | 61 (23.6) | 78 (26.3) | 19 (19.0) | 6 (15.4) | 0.37 |
| Hypertension (%) | 524 (71.1) | 11 (68.8) | 173 (64.6) | 218 (71.0) | 89 (86.4) | 33 (76.7) | 0.001 |
| Systolic BP mmHg | 156 | 150 | 157 | 156 | 154 | 160 | 0.492 |
| (median [IQR]) | [13, 17] | [132, 174] | [136, 175] | [140, 172] | [139, 170] | [148, 178] |  |
| CHD (%) | 124 (16.9) | 3 (18.8) | 40 (14.9) | 54 (17.6) | 18 (17.6) | 9 (21.4) | 0.819 |
| Diabetes mellitus, n (%) | 133 (18.2) | 2 (12.5) | 36 (13.5) | 48 (15.6) | 30 (30.0) | 17 (40.5) | <0.001 |
| Blood glucose in mmol/L | 6.4 | 6.05 | 6.4 | 6.4 | 6.65 | 7 | 0.013 |
| (median [IQR]) | [5.60, 7.68] | [5.47, 7.48] | [5.70, 7.53] | [5.60, 7.50] | [5.70, 7.82] | [6.20, 9.33] |  |
| Statin use (%), n(%) | 98 (25.5) | 1 (12.5) | 26 (20.2) | 45 (26.9) | 19 (32.2) | 7 (33.3) | 0.297 |
| Antithrombotic use, n (%) |  |  |  |  |  |  | 0.041 |
| Antiplatelets | 362 (40.7) | 7 (31.8) | 117 (36.6) | 160 (42.8) | 51 (41.8) | 27 (51.9) |  |
| Anticoagulants | 42 (4.7) | 1 (4.5) | 9 (2.8) | 18 (4.8) | 11 (9.0) | 3 (5.8) |  |
| No antithrombotics | 486 (54.6) | 14 (63.6) | 194 (60.6) | 196 (52.4) | 60 (49.2) | 22 (42.3) |  |
| Atrial fibrillation (%) | 214 (29.5) | 8 (50.0) | 78 (29.5) | 90 (29.6) | 22 (22.0) | 16 (38.1) | 0.115 |
| NIHSS (median [IQR]) | 9 | 12 | 10 | 8 | 7.5 | 9 | 0.05 |
|  | [5.00, 15.00] | [8.75, 17.75] | [6.00, 16.00] | [5.00, 15.00] | [5.00, 14.00] | [6.00, 13.00] |  |
| Stroke onset to treatment time (minutes [IQR])) | 150 | 130 | 150 | 150 | 150 | 135 | 0.175 |
|  | [111, 182] | [91, 161] | [110, 180] | [118, 180] | [120, 205] | [110, 180] |  |
| Stroke etiology, n (%) |  |  |  |  |  |  | 0.946 |
| Large-artery atherosclerosis | 136 (15.6) | 4 (19.0) | 42 (13.4) | 59 (16.0) | 24 (19.8) | 7 (13.7) |  |
| Cardioembolism | 358 (41.0) | 11 (52.4) | 130 (41.5) | 148 (40.2) | 46 (38.0) | 23 (45.1) |  |
| Lacunar | 48 (5.5) | 0 (0.0) | 17 (5.4) | 20 (5.4) | 8 (6.6) | 3 (5.9) |  |
| Other | 58 (6.6) | 0 (0.0) | 21 (6.7) | 27 (7.3) | 8 (6.6) | 2 (3.9) |  |
| Undetermined | 274 (31.4) | 6 (28.6) | 103 (32.9) | 114 (31.0) | 35 (28.9) | 16 (31.4) |  |
