# Supplementary material for: No Impact of Body Mass Index on Outcome in Stroke Patients Treated with IV Thrombolysis BMI and IV Thrombolysis Outcome
Source: PLoS One. 2016 Oct 11;11(10):e0164413. doi: 10.1371/journal.pone.0164413 (PMC5058473; doi:10.1371/journal.pone.0164413)
Supplement: S2 Table — (DOCX) [file pone.0164413.s002.docx]

Supplemental material:

1. Patient numbers and study periods for each center

| **Center** | **Number of patients** | **Period** |
| --- | --- | --- |
| University Hospital Lausanne | 506 | 01/2003 – 11/2013 |
| University Hospital Zurich | 164 | 12/2009 – 09/2012 |
| Cantonal Hospital St.Gallen | 122 | 06/2010 – 08/2013 |
| University Hospital Berne | 104 | 10/2012 – 06/2014 |
